# Early Detection of Hepatocellular Carcinoma: How to Screen and Follow up Patients with Liver Cirrhosis According to the GERMAN S3 Guideline?

**DOI:** 10.3390/diagnostics5040497

**Published:** 2015-11-25

**Authors:** Ruben R. Plentz, Nisar P. Malek

**Affiliations:** Department of Internal Medicine I, Medical University Hospital, Otfried-Müller-Str. 10, 72076 Tübingen, Germany; E-Mail: nisar.malek@med.uni-tuebingen.de

**Keywords:** diagnostics, German S3 guideline, hepatocellular carcinoma

## Abstract

Hepatocellular carcinoma (HCC) is frequently detected in pre-existing liver cirrhosis, but can also develop without such pre-conditions. There is an increasing trend of HCC incidence worldwide. In patients with liver cirrhosis, HCC has become the leading cause of death. At diagnosis the tumor has very often reached an advanced stage and curative treatment options are missing. Thus, early diagnosis would help the patient and prevent increasing healthcare costs. In our review we will summarize the recommendations of the German S3 guideline for the early diagnosis of HCC and will discuss the current literature in this context. The reader will learn which diagnostic tools are available and in what order they can be usefully applied. Surveillance should be done with ultrasound by a skilled examiner, additional imaging at best with state-of-the-art dynamic magnetic resonance.

## 1. Introduction

**Figure 1:**
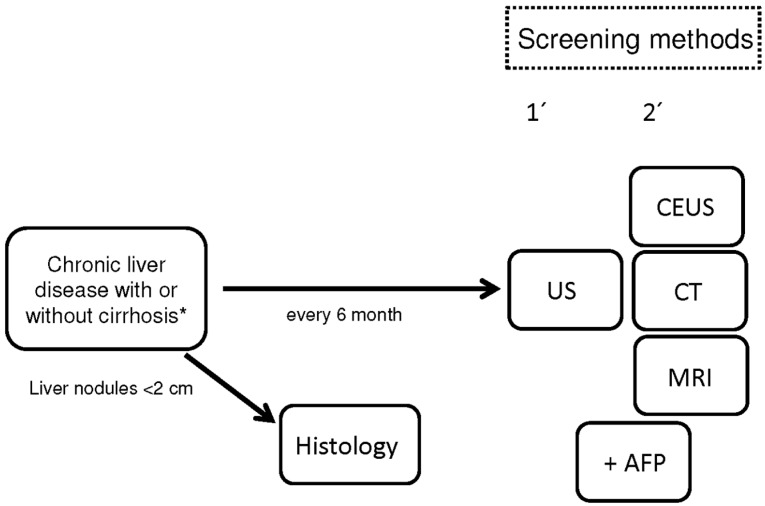
Screening test in patients with chronic liver disease or cirrhosis according to the German S3 guideline. AFP: alpha-fetoprotein, CEUS: contrast-enhanced ultrasound, CT: computer tomography, MRI: magnetic resonance imaging, US: ultrasound. * No patients with CHILD-Pugh Score C.

Patients with liver cirrhosis independent of their etiology and also individuals without cirrhosis but with chronic hepatitis B infection or non-alcoholic fatty liver disease (NAFLD) have a very high risk for the development of hepatocellular carcinoma (HCC) [1]. Worldwide, HCC is the fifth most common malignancy [2]. In Europe, North America, and Japan, the HCC incidence is lower compared to South-Asian countries and Africa, but not negligible [3]. Thus, a challenging task is the early diagnosis of HCC in patients with known risk factors. Patients with HCC and tumor symptoms have a survival rate of only 0%–10% [4]. In contrast, patients with an early diagnosis of HCC can achieve five-year survival rates of over 50% [1]. However, the best prevention strategy for HCC development is to avoid the development of liver disease and the progression to liver cirrhosis. Hepatitis B vaccination in children had led to a dramatic decrease of HCC incidence [5]. The treatment of alcohol disease is also very helpful to keep the HCC incidence low, and the reduction of body mass index will also help to decrease HCC development. According to the recently published German S3 guideline for HCC, it is recommended to perform regular screening tests in patients with liver cirrhosis if they would benefit from an early tumor diagnosis (Figure 1) [6]. However, only patients with compensated liver function will benefit from an early HCC diagnosis, since tumor therapies are not feasible in individuals with impaired liver function. An exception is made for patients waiting for a liver transplantation. However, patients without liver cirrhosis but with chronic liver diseases should be observed on a regular basis [7,8,9,10,11,12].

## 2. Imaging Techniques

The radiological characterization of HCC is based on its typical tumor perfusion patterns. Ultrasonography (US) as well as contrast-enhanced ultrasound (CEUS), contrast-enhanced computer tomography (CT), and magnetic resonance imaging (MRI) are available (Table 1). US is a non-invasive method, but it is dependent on the quality of the ultrasound device and the level of training of the investigator [13,14,15]. Abdominal US reached a specificity of over 90% and a sensitivity between 58% and 89% for the detection of HCC [16]. In a meta-analysis of 13 trials involving 2715 patients, in which US was used for early detection, a sensitivity and specificity of 94% for the preclinical detection of HCC was revealed [17]. For the first imaging in patients with liver disease, US should ideally be carried out by the same and well-qualified examiner. Quality-assured US is based on equipment quality and technology, documentation standards, and regular tests of acoustic probes and of the US device. With regular inspection intervals as well as the attention of the examiner, about 98% US device malfunctions were discovered in time [18]. Simpler and cheaper portable US devices have deficits in the diagnosis of small HCC lesions [19,20]. In addition to the B-mode scan, a color Doppler should be implemented in the device. For example, the proof of a portal vein thrombosis only by B-mode imaging is insufficient, but for the early diagnosis of HCC is quite essential. A portal vein thrombosis in a patient with liver cirrhosis is always highly suggestive of the presence of liver malignancy. Evidence of arterial vascularization of portal vein thrombosis proves the presence of HCC, even if in the B-mode scan no mass can be detected [21]. After detection of a new or suspicious liver lesion, additional contrast-based imaging is required, and CT or MRI are best suited to this purpose. The sensitivity and specificity of these methods are comparable [22,23]. CEUS mainly has a role in the characterization of small lesions in the liver in both cirrhotic and non-cirrhotic liver [24]. Arterial hypervascularization is the leading criterion for the diagnosis of HCC by CEUS. In addition, the sensitivity (66.6%, 87.5%, 91.7%, 97.3%) of US correlates with the tumor sizes (*n*): *n* ≤ 1 cm, 1 cm ˂ *n* ≤ 2 cm, 2 cm ˂ *n* ≤ 3 cm, *n* > 3 cm [25]. For early HCC detection, two randomized studies from China were published. In the first one, 18,816 patients with chronic hepatitis B infection regardless of cirrhosis status underwent US investigation and determination of the tumor marker alpha-fetoprotein (AFP) every six months and were compared to patients without any screening [26]. Although only 58% of the patients agreed to the screening methods, a reduction of 37% of HCC-related mortality was demonstrated by a combination of US and AFP. In the second study, AFP was measured every six months in a population of HbsAg-positive patients and compared to individuals without any screening [27]. By AFP measurement the detection of early HCC was high, but there was no difference in five-year survival and/or HCC mortality between the groups. The combination of US and AFP led only to an increase in sensitivity from 63% to 69%. Clinical trials on the usage of CT and MRI as a method for early HCC detection are missing; therefore, these techniques are not routinely recommended for screening purposes. However, an exception is made for patients with insufficient US conditions such as obesity, limited compliance, *etc.* HCC confirmation by CT requires evidence of a contrast tumor nodule with arterial hypervascularization and subsequent wash-out area [6,28]. In contrast, small dysplastic nodes (<2 cm) often cannot be distinguished from small HCCs by CT technology. For larger nodes (>2 cm), multifocal, and diffuse HCC, there is detection accuracy by CT above 80%. MRI allows an improved differential diagnosis of HCC-suspicious lesions, and new contrast agents such as Gadoxetate dimeglumine are available. These contrasts have demonstrated an improvement in distinguishing between small HCCs and benign liver lesions [29]. In a small study of patients waiting for liver transplantation, different imaging techniques were tested by two examiners. US performed by a skilled examiner showed the highest sensitivity with 89% compared to 67%/56% for CT, 56%/50% for MRI, and 0% for PET [30].

**Table 1:** Comparison of HCC Imaging Techniques.

Modality	Specificity	Sensitivity	Reference
US	90%	58%–89%	[16]
US	94%	94%	[17]
US	-	89%	[30]
US + AFP	-	69%	[27]
CEUS	-	66.6%–97.3%	[25]
CT	80%	80%	[6,28]
CT	-	56%–67%	[30]
MRI	-	50%–56%	[30]

The mean doubling time of HCCs lies between 140 and 200 days. Therefore, a screening interval of six months is recommended. In one study the screening interval of three months was tested, but did not show any advantage compared to the six-month period [31]. A meta-analysis of prospective studies revealed that the sensitivity with semiannual screening of 70% drops to 50% for annual screenings [17]. For these discussed reasons, it is recommended that the HCC screening be carried out on a semiannual basis by US. For suspicious liver nodules <2 cm without typical contrast characteristics in the initial imaging, histology reached the highest specificity and should be used primarily for diagnostic clarification [32]. Nuclear medicine examination methods such as FDG-PET-CT currently have no significance in early HCC diagnosis. Also, the importance of other serological markers such as Des-gamma-carboxy prothrombin-(DGCP) or fractions of AFP (AFP-L3) are not established yet.

## 3. Conclusions

In the future it would be desirable to have an even more “personal diagnostic algorithm”. Upcoming questions are: Should the prevention of patients with different liver diseases be different? Do patients with NAFLD/NASH (non-alcoholic steatohepatitis) need a different observation method compared to viral inflammation? Is there a distinction between cirrhosis and non-cirrhosis patients? Would a new biomarker help the pathologist and clinician to make an early diagnosis of HCC [33]? Can new imaging techniques, e.g., MRI and diffusion-weighted imaging, provide a higher resolution or better detection of especially small HCC lesions? However, it might be useful to combine different panels of biomarkers and imaging to predict high accuracy of HCC for early detection, but future studies are needed to provide more clarity.
